# Implications of oil price shocks on net oil-importing African countries

**DOI:** 10.1016/j.heliyon.2019.e02208

**Published:** 2019-08-28

**Authors:** Obindah Gershon, Nnaemeka Emmanuel Ezenwa, Romanus Osabohien

**Affiliations:** a Department of Economics and Development Studies, Covenant University, Ota, Nigeria; b Centre for Economic Policy and Development Research (CEPDeR), Covenant University, Ota, Nigeria; c Emerald Energy Institute, University of Port Harcourt, Choba, Rivers State, Nigeria

**Keywords:** Economics, Oil-importing countries, Oil price shocks, GDP per capita

## Abstract

The study examines the implications of oil price shocks on developing net oil-importing countries. The study considers the casual relationship, impulse response function, and vector decomposition between oil prices and macroeconomic variables using an unrestricted vector autoregressive (VAR) model. In addition, other robust econometric techniques were applied to the time series of oil prices, GDP per capita (GDPC), and energy consumption from 1980 to 2015. Mix results were obtained for the selected African countries - Cape Verde, Liberia, Sierra Leone, and The Gambia. Evidence from the granger test shows that oil prices cause GDPC in Liberia and Sierra Leone. Furthermore, analyses from the VAR model and Impulse response indicate that oil price increase will temporarily increase GDP per capita in the short run for the selected countries. The study recommends policies that can effectively mitigate the adverse effect of the oil price increase.

## 1 Introduction

Oil price fluctuations affect economies differently depending on whether they are net oil-importers or net oil-exporters. High oil prices for net oil-importing countries could lead to high import costs with an adverse effect on GDP, exchange rate, inflation and balance of payment. However, high oil prices for net oil-exporters improves the general balance of payment due to the increase in oil revenue. In addition, high oil price volatility increases uncertainty regarding cash flows which can be challenging for government in policy decisions.

The Arab oil embargo of 1973–1974, which caused the first oil shock, has triggered several discussions on the causal link between oil price and macroeconomic activities. Recent studies of this relationship on small oil-importing countries (Abimelech et al., 2017), claimed that rising oil prices will stimulate economic growth, which is not consistent with other studies that rising oil prices have an adverse effect on net oil-importers (Yanagisawa, 2012; Lemazoshvili, 2014; Shabhaz et al., 2017).

The development of the global energy market dating back from 1970 period has been dramatic with significant impact on global economy and politics. This is observed by the global economic shocks caused by the price fluctuations of the most consumed energy resource which is oil. Global crude oil prices have recently been on increase due to the cuts by the oil cartel OPEC, led by Saudi Arabia and Russia that has balance the oil market forces of demand and supply. In July 2014 the average price for Brent was $45 USD per barrel rose up to $80 USD per barrel in May 2018 according to Adam (2018). Demand keeps on increasing in the Western world and in the Middle East such as Brazil, China, and India etc. The U.S. Energy Information Administration (2017) forecast world energy consumption to grow by 28% between 2015 and 2040. The rising of energy prices and the anticipated increase in global consumption growth is likely to place net-importing economies, especially Africa in a tight position.

Several empirical studies on the impact of oil price shocks on economic components have been conducted for oil-importing developed economies such as United States, European Union, and Japan as in (Kurihara, 2015) and (Akira, 2012), where oil price increase causes economic growth. However, studies have examined oil-importing developing countries such as Bangladesh, El Salvador, Kenya, Nicaragua, Tanzania and Thailand in (Sachez, 2011). The increase in oil price generally negative impact in GDP causing increase in unemployment, higher consumer prices and reduced welfare for citizens.

Studies have analysed the impact of oil price shocks on developed and developing economies, although there is scarce research of this nature targeted at high indebted poor countries. Therefore, the aim of the research is to investigate the effects of oil price shock on oil-importing poor West African countries. The paper selected three low-income countries (Sierra Leone, Liberia and The Gambia) out of the 39 countries listed in the IMF debt relief initiative (IMF, 2016) and a low-middle income country, Cape Verde (The World Bank, 2018a, The World Bank, 2018b). The Gambia, Sierra Leone, and Liberia are categorized as highly indebted poor countries (HIPC) according to The World Bank, 2018a, The World Bank, 2018b. The World Bank group in collaboration with International Monetary Fund (IMF), and African Development Bank are seeking to end extreme poverty by 2030. This is an important study for poverty alleviation for organisations, and governments to mitigate the possible implications of the global oil price shock on the key macroeconomic components of net-oil importing poor countries in order to improve the balance of payment and manage debt crises.

The paper contributes to knowledge by highlighting policies that can maximize the positive impacts and mitigate the negative effect of the net oil-importing poor countries. The macroeconomic framework of the selected countries varies in respect to the stylized facts: net import of oil per GDP, oil dependence in the energy mix, efficiency of energy production, and level of exports, revenues and savings from international trade.

## 2 Background

### 2.1 Literature review

Studies have discussed energy prices, GDP and energy consumption relationship in different economies. Osigwe and Arawomo (2015) examined the granger causality of the variables under the error correction model. The empirical evidence suggested that bi-directional causality exist between electricity consumption and economic growth, Similar to Matthew et al. (2018). Also, electricity prices and electricity consumption are found to be bi-directional. Moreover, the literature suggests the implementation of energy pricing model that can promote consumption as well as economic output. Shengfeng et al. (2012) via the vector error correction model (VECM) investigated the short-run and long-run causal relationship between electricity consumption and economic growth in China. The study observed unidirectional causality from electrical consumption to GDP growth and suggested the need for consumers to maintain regular supply through saving that fosters economic growth.

Bekhet and Yusop (2009) investigates that long-run relationship exists between oil prices, energy consumption, GDP, employment, and population in Malaysia. The results shows negative relationship between GDP growth and energy consumption and positive relationship between energy consumption and economic growth. The study suggested that reducing the consumption of fossil fuel and moving tos hydropower and biomass will have positive effect on the current account balance. There have been several studies on the oil price – the macroeconomic link that involves different modelling methods.

Some studies implement the general method of moments (GMM), while others combine or only employ the various econometric techniques. For instance, Alley et al. (2014) implemented the GMM model to investigate the impact of oil price shocks on the Nigerian economic activities. The study shows insignificant oil price shocks in time of economic uncertainty that slows down the economy and rising oil prices resulting to economic growth. The study also presents cointegration analysis using the Johansen system of cointegration, which suggest no long-run relationship between oil prices and GDP.

Ahmed and Moran (2013) use the momentum threshold auto-regressive model to examine the long-run relationship and the existence of asymmetry between real oil prices and the real exchange rate of twelve oil exporters. They also employ the augmented the Dickey-Fuller test (ADF) and garner causality to test for the presence of unit root which determines the level of integration and causality of variables respectively. The results indicate the presence of co-integration in six out of twelve economies studied. The results also reveal the asymmetry adjustments in Nigeria, Brazil, the United Kingdom, and the Eurozone. Brazil and the UK exchange rate indicated granger causality with oil prices.

Schubert and Turnovsky (2011) investigate the effects of an oil price increase on the long-run growth and output performance of a small oil-importing developing economy. The study implements a dynamic stochastic model and restricts access to the small oil-importing developing economy from the borrowing costs. The results suggest that the long-run effects of oil price increase can be determined basically by internal production conditions which are the relative share of labour in input and the elasticity of substitution in production. Idrisov et al. (2015) use a DSGE model to observe that an increase in oil prices cannot impact the long-term economic growth rate but can be determined by the efficiency of production factors. The influence of oil prices on the GDP may be observed only from a short-run dynamics. Also, the study observes a positive relationship between oil prices and the GDP of Russia in the short-run. The economic boom is caused by controlling the nominal rate of the Russian Ruble, which improves the trade balance. The short-term impact of oil price changes to GDP is more significant than the long-term contribution.

Various authors have adopted the vector autoregressive (VAR) model with various cointegration techniques. Lemazoshvili (2014) study the impact of oil price shocks on oil-importing small open economies using Armenia and Georgia as a case study. They use the structural VAR to show oil price shocks directed impact the Georgian economy through imported oil refined products and level of oil consumption in the transportation sector. Also, in the Armenian economy, the natural gas pricing seems to counter the effect of oil price shocks. Muhammad and Ghulam (2017) use the VAR model to investigate the effect of oil price volatility on GDP and the trade balance of Pakistan. They conclude the stable effects of oil shocks on variables, and variables do not influence each other. Yukata (2015) investigates the relationship between oil prices and economic growth in developed countries. The results of this relationship from the theoretical and empirical perspective are unclear. Also, oil price increase benefiteconomic output in the US, EU, and Japan.

In light of the literature review, there are diverse conclusions from several authors on various economies. Hence, we study oil price shocks and their impacts on the macroeconomic components of oil-importing West African Economies: The Gambia, Liberia, Sierra Leone and Cape Verde.

## 3 Methodology

The vector autoregressive (VAR) model is an important model for analysing the dynamic behaviour of economic time series and forecasting. The natural response of the economy to macroeconomic shocks and the contribution of these shocks in the formulation of macroeconomic framework is incorporated in the VAR models. The model provides advanced forecast to those from univariate time series models and theoretical simultaneous equation models. The VAR model is flexible in predicting the future interaction of variables (Fomby et al., 2013). The vector autoregressive model (VAR) is a multivariate time series model which treats all variable series endogenously and the estimation uses the past values of the dependent variables and other variables. The fundamental systems of equations used for the vector autoregressive or short-run model in the reduced form are given below:

(1) $$\ln O_{t}=C_{1}+\alpha_{i}^{1}\overset{n}{\underset{i=1}{\sum }}\ln O_{t-i}+\beta_{j}^{1}\overset{n}{\underset{j=1}{\sum }}\ln\;GDPC_{t-j}+\theta_{m}^{1}\overset{n}{\underset{m=1}{\sum }}\ln\;EC_{t-m}+\mu_{t}^{1}$$

(2) $$\ln\;GDPC_{t}=C_{2}+\alpha_{i}^{2}\overset{n}{\underset{i=1}{\sum }}\ln O_{t-i}+\beta_{j}^{2}\overset{n}{\underset{j=1}{\sum }}\ln\;GDPC_{t-j}+\theta_{m}^{2}\overset{n}{\underset{m=1}{\sum }}\ln\;EC_{t-m}+\mu_{t}^{2}$$

(3) $$\ln\;EC_{t}=C_{3}+\alpha_{i}^{3}\overset{n}{\underset{i=1}{\sum }}\ln O_{t-i}+\beta_{j}^{3}\overset{n}{\underset{j=1}{\sum }}\ln\;GDPC_{t-j}+\theta_{m}^{3}\overset{n}{\underset{m=1}{\sum }}\ln\;EC_{t-m}+\mu_{t}^{3}$$

Where: O, GDPC, and EC represent oil prices, GDP per capita and energy consumption respectively, C is constant term α,β,θ are the variable coefficient, and μ is the white noise. Following the study of Dechassa et al. (2017) the basic VAR model consist of a set ofK macroeconomic indicators assuming that $Y_{t}=\;\left\{O,\;GDPC,\;EC\right\}$ represent a vector of n variables at time t.

The VAR model considers the following assumptions: (i) stationarity, (ii) $\mu_{t}$ is an n-dimensional white noise process with $E\left[\mu_{t}\right]=\;0,\;\;\omega =\left(\omega_{1},\;\omega_{2},\;\ldots ,\;\omega_{n}\right)$ is an (n×1) vector of the coefficient which allows for a non-zero mean, $E\left(Y_{t}\right)$, $\mu_{t}$ is the positive covariance matrix of serially uncorrelated error terms.

### 3.1 Unit-root test

The unit root testing deals with the underlying statistical properties of the individual variables in the VAR model. A variable is valid in the regression model if it has a constant mean, constant variance, and constant co-variance. The fundamental assumption is that the series in the regression model must be predictable or stationary. The eligibility of the individual variables can be evaluated through unit root testing. There are two prominent used techniques to test the stationarity of a time series variable: Augmented Dickey and Fuller (ADF) (1979) and Phillips and Perron (PP) (1988) unit root tests.

The Augmented Dickey Fuller tests for unit root in the AR(1) model. The Phillips-Perron (PP) test is an extension of the ADF test that corrects any serial correlation that occur by adding lagged differences of the residual in the regression model. The underlying hypothesis are:

$$H_{o}:There\;is\;unit\;root$$

$$H_{1}:There\;is\;no\;unit\;root$$

A rejection of $H_{o}$ implies that the series is stationary, otherwise it is not stationary. The series is said to be I(0) stationary, if there is evidence of stationairty at the level series. Empirically, if a variable is I(0), it does not require any transformation since it is predictable and stable. However, if a variable is non-stationary, the transformation is required by first differencing to achieve stationarity. i.e. Oil price $\left(O_{t}\right)$

$$O_{t}∼I\left(0\right)\to No\;transformation\;required$$

$$if\;O_{t}∼I\;\left(d>0\right)\to Transformation\;is\;required$$

$$O_{t}-O_{t-1}=\Delta O_{t}∼I\left(1\right)=first\;difference$$

### 3.2 Co-integration test

Co-integration test is needed when the variables are not stationary at levels. If the time series variables are stationary at levels, then co-integration test is not required. This shows that the long run effect is not different from the short run effect. Series that are non-stationary that become stationary when first differenced, is said to be integrated of order 1. Hence, is essential to test for co-integration among variables to ascertain the existence of long run effects.

Two prominent approaches for the co-integration test are the Engle and Granger (1987) two step procedure and Johansen (1988) maximum likelihood procedure. The Johansen approach is employed for this study. The approach uses the maximum likelihood technique of the VAR model to determine the number of r, co-integrating vectors which is usually accounted by the two likelihood ratio (LR) test statistics namely; Trace test and Max Eigen value statistics. This approach investigates for long term relationship between variables in a multi-variate system. The underlying hypotheses are:

$$H_{o}:r=0\;\Rightarrow No\;cointegration\;\Rightarrow No\;long\;run\;relationship$$

$$H_{1}:r\neq 0\Rightarrow Cointegration\;\;exists\Rightarrow long\;run\;relationship\;exist$$

### 3.3 Data sources

**Fig. 1:**
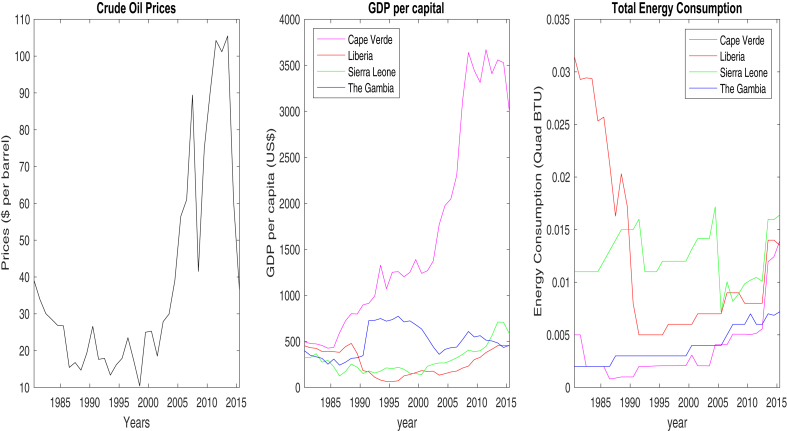
Average Crude oil prices (WTI, Brent, Dubai Crude), GDP per capita, Total Energy Consumption. Source: Authors.

The empirical study uses the average of three major oil benchmark prices; West Texas Intermediate, Brent Blend, and the Dubai Crude spot prices from (Quandl, 2018) as a proxy for global oil price. The macroeconomic variables for the four net oil-importing West African countries (Cape Verde, Liberia, Sierra Leone, The Gambia) considered in the investigation are GDP per capita measured in US dollars and total energy consumption measured in quadrillion BTU. Data for GDP per capita (GDPC) and total energy consumption (EC) was obtained from (The World Bank, 2018a, The World Bank, 2018b) and (U.S. Energy Information Administration, 2015) websites respectively. The data series are yearly observation from 1980 to 2015, a total of 36 observations. The period and the frequency of the selected dataset adopted for the investigation is based on data availability. Fig. 1 shows the time series for the average oil price, GDP per capita and total energy consumption for the four developing net-oil importing countries.

### 3.4 Descriptive statistics

The statistical analysis of the data series on E-VIEWS 10 in Table 1 shows the mean, standard deviation, skewness, kurtosis, and Jarque-Bera. We use the linear form of the dataset to compare the moments of the distribution of the data series. The first and second moment of the distribution suggests that the sample mean is not zero and the standard deviation lie in the range of 0.2031 and 0.7157. The most volatile series is the total energy consumption of Cape Verde and the least volatile series is the GDP per capita (GDPC) of The Gambian economy. The time series of Global oil price, the energy consumption (EC) distribution of Cape Verde, Liberia, The Gambia, and Sierra Leonean GDPC are skewed to the right, while the Cape Verdean, Liberian, The Gambian GDPC and Sierra Leonean EC are skewed to the left. There is asymmetry in the probability distribution of the variables about their mean.

**Table 1:** Descriptive Statistics of variables.

Countries	Variables (log)	Mean	Std. Dev	Skewness	Kurtosis	Jarque-Bera
Global	OIL	3.4427	0.6469	0.5027	2.2214	2.4257
CAPE VERDE	GDP per Capita	7.1854	0.7060	−0.0063	1.8653	1.9314
	Energy Consumption	−5.8779	0.7157	0.4068	2.7862	1.0615
LIBERIA	GDP per Capita	5.4334	0.6231	−0.4959	2.1013	2.6868
	Energy Consumption	−5.6352	0.4178	0.2195	1.9432	1.9641
SIERRA LEONE	GDP per Capita	5.5910	0.4541	0.4332	2.5202	1.4711
	Energy Consumption	−4.4149	0.2031	−0.2893	2.8022	0.5607
THE GAMBIA	GDP per Capita	6.1535	0.3451	−0.1732	1.8997	1.9960
	Energy Consumption	−4.5916	0.6116	0.6226	1.9668	3.9270

Source: Authors.

### 3.5 Stationarity analysis

The methodology which adapts the vector autoregressive model (VAR) requires that the variables have to be trend stationary. Generally, it has been observed in studies that most of the data series are not stationary at level I(0), but attain stationarity after first differencing I(1) (Abimelech et al., 2017) and (Lemazoshvili, 2014).

The study to determine the unit root makes use of both the augmented Dickey-Fuller test (ADF) and Phillips-Perron Test. Table 2 shows the report of stationarity analysis carried out on the level I(0) and the first difference I(1) state. We observe from the level of significance, the rejection of the null hypothesis for the trend stationary model for most variables after taking the first difference. This allows for the study to implement the vector autoregressive model.

**Table 2:** Augmented Dickey-Fuller (ADF) and Phillips-Perron unit root test.

		Augmented Dickey-Fuller Test				Phillips-Perron Test				
Countries	Variable	Level		First difference			Level		First difference	
		None	Constant	None	Constant		None	Constant	None	Constant
Global	OIL	−0.38	−1.92	−1.80*	−1.68		−0.97	−1.60	−6.44***	−6.34***
CAPE VERDE	GDP per Capita	1.17	−0.48	−4.10***	−4.35***		−4.14	−0.56	−4.28**	−4.28**
	Energy Consumption	1.63	1.15	−5.15***	−5.26***		1.73	1.34	−5.19	−5.26***
LIBERIA	GDP per Capita	−0.65	−1.58	−3.11**	−3.06*		−0.63	−1.38	−3.57***	−3.52**
	Energy Consumption	3.53	1.75	−0.66	−3.65**		1.92	0.30	−5.85***	−6.55***
SIERRA LEONE	GDP per Capita	1.05	0.28	−4.25***	−4.23**		0.65	−0.38	−3.89**	−3.80***
	Energy Consumption	0.22	−2.94	−8.31***	−8.24***		0.08	−2.99	−8.28***	−8.21***
GAMBIA	GDP per Capita	−0.34	−1.50	−5.21***	−5.14***		−0.42	−1.75	−5.23***	−5.16***
	Energy Consumption	−1.79	−2.27	−4.80***	−4.86***		−2.42	−2.49	−4.81***	−4.85***

***, **, * Indicates the significant levels at 1%, 5%, and 10% respectively.

Source: Authors.

### 3.6 Co-integration analysis

The study tests for co-integration or long-run relationship among variables using the Johansen method. The test is performed on the level log form state of the variables and not on their first difference to ascertain if there is co-integration among variables as explained in (Gerald, 2015). Table 3 reports co-integration results of among variables for each oil importing countries.

**Table 3:** Johansen Co-integration test.

Hypothesized coefficients	No of Eigenvalue	T-statistics	Critical value (5%)	Probability
CAPE VERDE				
None	0.47125	28.93431	29.79707	0.0626
At most 1	0.136745	7.268136	15.49471	0.5467
At most 2	0.064546	2.26859	3.841466	0.132
LIBERIA				
None	0.353256	20.35228	29.79707	0.3992
At most 1	0.142417	5.534912	15.49471	0.7497
At most 2	0.009113	0.311262	3.841466	0.5769
SIERRA LEONE				
None*	0.464568	30.28648	29.79707	0.0439
At most 1	0.163674	9.047308	15.49471	0.361
At most 2	0.083654	2.970265	3.841466	0.0848
THE GAMBIA				
None	0.316658	21.32142	29.79707	0.3379
At most 1	0.146129	8.375574	15.49471	0.4261
At most 2	0.084574	3.004429	3.841466	0.083

*Indicates there is a co-integration.

Source: Authors.

The report shows that there are no co-integration among the investigated variables global oil prices, GDP per capita, and energy consumption for Cape Verde, Liberia, and The Gambia. However, there is co-integration in Sierra Leone as the t-statistics is greater than the critical value at 5% significance level. The exhibition of a long-run relationship among variables suggest that there are shocks even in the short-run which may affect movement in the individual series which converges with time in the long-run.

Estimation of series in Sierra Leone can be done using a short-run model which is the vector autoregressive (VAR) or the vector error correction (VEC) model to obtain both the short-run and long-run estimates. However, no co-integration among series variables for Cape Verde, Liberia, and The Gambia. This suggest that the estimation can only be done using the VAR model. Hence, for the purpose of our study to investigate and compare similar net oil-importing West African economies, we only estimate the short-run model using the unrestricted vector autoregressive model.

## 4 Results and discussion

### 4.1 Model lag selection criteria

The unrestricted VAR model requires that the optimum lag is selected using any of the criteria in EVIEWS 10. In theory, the most used method to select the lag order p is the information criterion procedure. An information criterion suggests the best model fit for the group of data from a set of observed models. The choice of how many lag order is needed in the linear regression model is reliant upon the model selection criterion. This is achieved by minimizing Akaike Information Criterion (AIC) or Schwartz Information Criterion (SIC) or Hannan-Quinn Information Criterion.

Table 4 shows the result of different lag selection criteria under the unrestricted VAR approach. The Akaike Information Criterion (AIC) as a method of lag selection suggest that lag 1 has the lowest AIC value for Cape Verde, Liberia, Sierra Leone, and The Gambia, and therefore should be best for the study. Other selection criteria for the selected net oil-importing countries also suggested that Lag 1 is the best lag for the study. Model lag selection is very important to be considered because the model will provide the best result needed to analyse the impulse responses of oil shocks and macroeconomic variables for each country.

**Table 4:** Unrestricted vector autoregressive lag selection criteria.

Lag	LogL	LR	FPE	AIC	SC	HQ
CAPE VERDE						
0	−71.12703	NA	0.015715	4.360413	4.495092	4.406343
1	17.62633	156.6236*	0.000145*	−0.330961*	0.207755*	−0.147243*
2	19.0489	2.259373	0.000229	0.114771	1.057523	0.436276
LIBERIA						
0	−64.38682	NA	0.010571	3.963931	4.09861	4.00986
1	29.05768	164.9021*	7.38e-05*	−1.003393*	−0.464677*	−0.819675*
2	37.72902	13.77213	7.64E-05	−0.98406	−0.041308	−0.662554
SIERRA LEONE						
0	−28.80844	NA	0.001304	1.871085	2.005763	1.917014
1	11.895	71.82959*	0.000203*	0.006177*	0.544892*	0.189894*
2	17.6505	9.141095	0.000249	0.197029	1.139781	0.518535
THE GAMBIA						
0	−52.95731	NA	0.005397	3.291607	3.426286	3.337536
1	19.94881	128.6579*	0.000126*	−0.467577*	0.071139*	−0.283859*
2	27.07863	11.32384	0.000143	−0.357567	0.585186	−0.036061

*Indicates lag order selected by the criterion.

LR: sequential modified LR test statistic (each test at 5% level), FPE: Final prediction error, AIC: Akaike information criterion, SC: Schwarz information criterion and HQ: Hannan-Quinn information criterion.

Source: Authors.

### 4.2 Granger causality analysis

The study employs the granger causality test to examine whether the past values of a variable help in predicting current changes in the unrestricted VAR model (Granger, 1969). Table 5 displays the results of the VAR Granger causality results for the net-oil importing countries.

**Table 5:** Short-run Granger causality results.

		CAPE VERDE	LIBERIA	SIERRA LEONE	THE GAMBIA
Dependent Variable (Log): OIL Chi – square					
Independent Variables (LOG)	GDP per capita	4.4668**	0.0534	0.9320	0.5090
	Energy consumption	0.1550	2.6695	0.9000	1.3567
Dependent Variable (Log): GDP per capita					
Independent Variables (Log)	Oil	1.2689	4.3792**	8.4519***	0.0010
	Energy Consumption	3.2087*	0.5125	0.0106	11.4239***
Dependent Variable (Log): Energy Consumption					
Independent Variables (Log)	Oil	1.8758	0.2859	0.6006	3.4415*
	GDP per capita	11.5686***	0.0015	0.5127	0.6846

***, **, * Indicates the significant levels at 1%, 5%, and 10% respectively.

The results when GDPC is the dependent variable shows that global oil price causes GDPC at 5% and 1% significance level for Liberia and Sierra Leone respectively, and has no influence on the Cape Verdean and The Gambian economy. Total energy consumption granger causes the GDPC of Cape Verde and The Gambia Economy at 1% and 10% significant level respectively. The amount of energy consumed affects the output and welfare of the people in these countries.

The estimates for energy consumption (EC) suggest that global oil prices granger causes EC for The Gambia at 10% significance level, while no causality effect for other oil importing countries. The GDPC's for Liberia, Sierra Leone, and The Gambia do not Granger causes EC, but the GDPC for Cape Verde influences energy consumption at 1% significance level.

### 4.3 VAR estimates

Table 6 presents the results of the unrestricted vector autoregressive model relative to the magnitude of the variables, and the predictive power. We explain the results that are significant at 5% threshold level.

**Table 6:** Unrestricted VAR results.

	CAPE VERDE			Liberia			Sierra Leone			The Gambia		
C. (No)	Coeff.	Std.	Prob.	Coeff.	Std.	Prob.	Coeff.	Std.	Prob.	Coeff.	Std.	Prob.
1	0.7331	0.1528	0.0000	0.6877	0.1502	0.0000	0.6901	0.1723	0.0001	0.8639	0.0911	0.0000
2	0.2388	0.1130	0.0372	0.0262	0.1133	0.8177	0.2359	0.2444	0.3368	−0.2120	0.2971	0.4774
3	−0.0585	0.1487	0.6947	0.3617	0.2214	0.1057	−0.3090	0.3257	0.3452	−0.1963	0.1685	0.2471
4	−1.1415	1.5685	0.4686	2.9756	1.4834	0.0478	−1.6170	1.7569	0.3598	0.8677	1.3154	0.5111
5	0.0636	0.0565	0.2629	0.1859	0.0889	0.0391	0.2819	0.0970	0.0046	−0.0013	0.0390	0.9738
6	0.9980	0.0418	0.0000	0.8613	0.0670	0.0000	0.5791	0.1375	0.0001	0.5224	0.1274	0.0001
7	−0.0985	0.0550	0.0765	−0.0938	0.1310	0.4758	0.0189	0.1832	0.9179	−0.2442	0.0722	0.0011
8	−0.7367	0.5798	0.2071	−0.4187	0.8775	0.6344	1.4748	0.9886	0.1391	1.8242	0.5638	0.0017
9	0.1702	0.1242	0.1741	0.0252	0.0471	0.5941	−0.0706	0.0911	0.4403	0.0940	0.0507	0.0667
10	0.3125	0.0919	0.0010	−0.0014	0.0355	0.9691	0.0925	0.1292	0.4758	0.1367	0.1653	0.4101
11	0.6088	0.1209	0.0000	0.9541	0.0694	0.0000	0.4819	0.1722	0.0062	0.9519	0.0937	0.0000
12	−5.1114	1.2752	0.0001	−0.3018	0.4647	0.5176	−2.5530	0.9288	0.0072	−1.4100	0.7316	0.0570
$R^{2}$	0..7658	0.9716	0.8714	0.7547	0.9045	0.9391	0.7444	0.8354	0.2734	0.7443	0.8345	0.9020
$Adj\;R^{2}$	0.7430	0.9689	0.8590	0.7310	0.8952	0.9332	0.7196	0.8195	0.2031	0.7196	0.8185	0.8925
D-W stat	2.1371	2.7360	2.1887	2.0827	1.2745	2.1274	2.0193	1.9311	2.2017	2.3266	1.5012	1.6583

Source: Authors.

There is a negative relationship between energy consumption (EC) and GDPC of Cape Verde when GDPC is the dependent variable. An increase in energy consumption by 1 quadrillion BTU will lead to a decrease in 0.0985 USD of GDPC. Energy consumption is a good predictor for the GDPC for Cape Verde. The slope coefficient is significant at 5% level.

There is a positive relationship between global oil prices and GDPC when GDPC is the dependent variable for Liberia. Thus, is observed that an increase in 1 USD per barrel of global oil price will result to an increase 0.1859 USD of GDPC. The slope coefficient is statistically significant at 5% level. Also, for Sierra Leone, there is also a positive relationship between global oil prices and GDPC when GDPC is the dependent variable. An increase in 1 USD per barrel of global oil price will result to $0.2819 USD increase in GDPC.

There is a negative relationship between EC and GDPC when GDPC is the dependent variables for The Gambia. An increase in EC by 1 Quad BTU will result in a $0.2442 USD decrease in GDPC. The slope coefficient is significant at 1% level. In addition, there is a positive increase between global oil price and EC when EC is the dependent variable. An increase in oil price by 1USD per barrel will result in 0.0940 Quad BTU in energy consumption.

The adjusted R-squared $\left(R^{2}\right)$ for the 12 systems generated by the unrestricted VAR model suggests high predictive power of the model to which is more than 70%, while the unexplained component is below 30%. Judging by the Durbin Watson (DW) value which for most of the systems are approximately 2, shows that there is no evidence of serial correlation. The systems where the Durbin Watson value is less than 2, is also less than the $R^{2}$ value, indicating no spurious relation may be evident.

### 4.4 Impulse response function

The impulse response function is an essential tool for policy analysis which traces the reaction of a variable for a specified period after a one standard deviation shock has occurred to another variable or itself. Instantaneous correlation of the error terms may occur separately which allows the use of the Cholesky decomposition technique to factorize the variance-covariance matrix under the vector autoregressive framework (Abimelech et al., 2017).

**Fig. 2:**
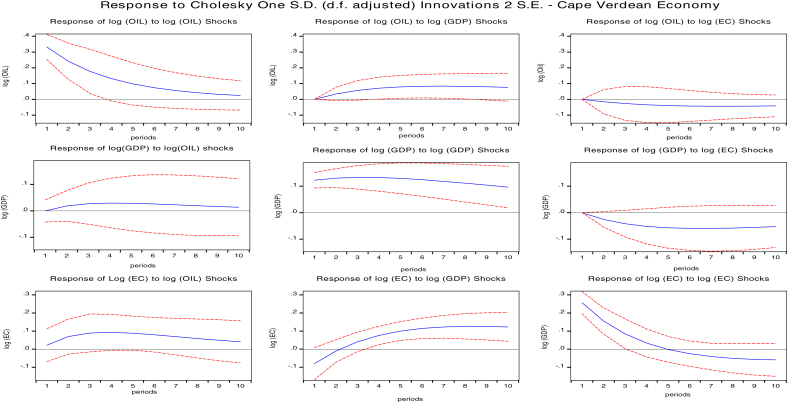
Impulse response function of shocks for Cape Verde. Source: Authors.

**Fig. 3:**
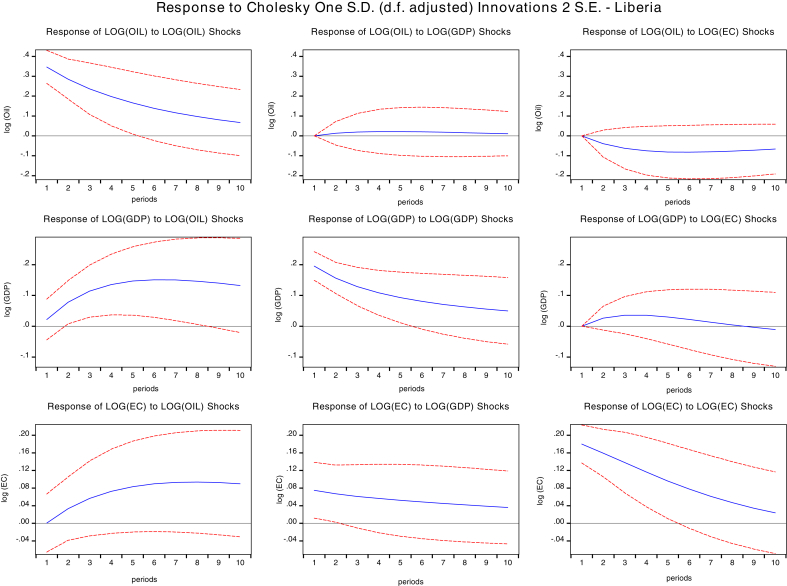
Impulse response functions for Liberia. Source: Authors.

**Fig. 4:**
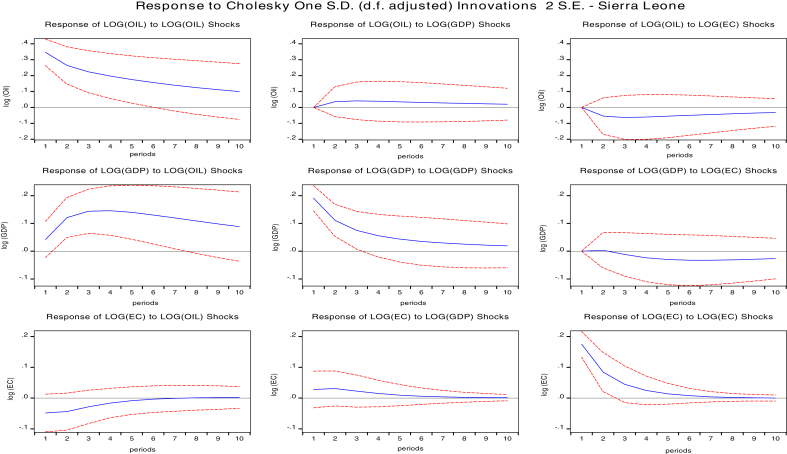
Impulse Response functions for Sierra Leone. Source: Authors.

**Fig. 5:**
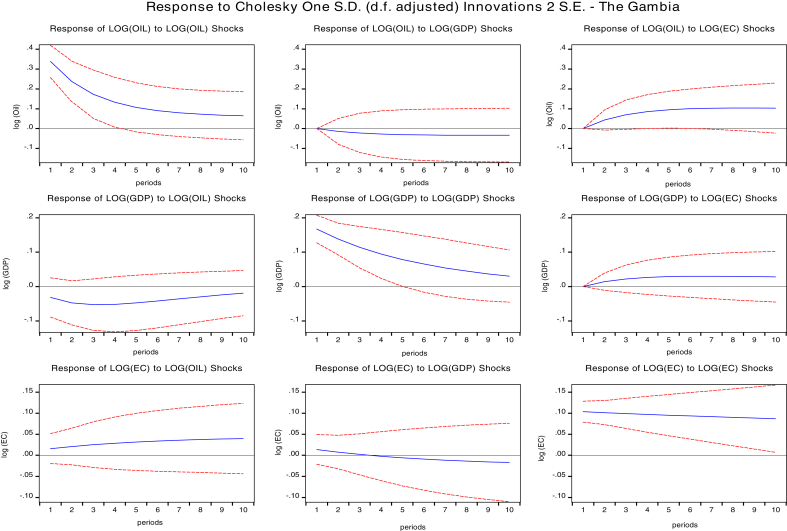
Impulse response function for Gambia. Source: Authors.

We presented the result of impulse responses in Figs. 2, 3, 4, and 5 for 10 years forecast period. A one standard deviation shock to global oil prices temporary increases GDPC of Cape Verde, Liberia Sierra Leone, and The Gambia. This positive response gradually falls after the fourth year for Cape Verde and Liberia, and in the fifth year for Sierra Leone. Beyond these periods, GDPC rises above slightly then after the third year decreases gradually and remains in the positive region. Global oil price shocks generally have a positive impact on the GDP per capita of both Cape Verde and Liberia in the short-run (1–3 years) and long run (7–10 years). However, oil price shocks to GDPC of The Gambia lie in the negative region. The positive response declines after the third year then reach a steady state in the long-run. Therefore, oil shocks to The Gambia's GDPC will have a negative impact on GDPC in the long-run and short-run.

Response of shocks to energy consumption (EC) when one std. deviation of oil shock for Cape Verde, Liberia, and The Gambia are positive. The positive response on Cape Verde's EC declines gradually after the fourth period and remains in the positive region. The positive response on Liberia's EC attains a steady state in the positive region after the fourth year. The positive response on Liberia's EC attains a steady state in the positive region after the sixth year. In general, there is a positive impact of oil prices to the economic consumption of Cape Verde, Liberia, and The Gambia both in the short-run and in the long-run. There is also a positive impact on Sierra Leone's EC although the responses are majorly negative in the short-run while in the long-run global oil prices has no significant impact on EC.

We also studied the response of GDP per capita to a one standard deviation shock of energy consumption (EC) for the selected oil importing countries. The response of GDPC to EC are negative for Cape Verde, Sierra Leone, and the Gambia. However, for Liberia impacts are negative in the short-run and positive in the long-run. A one std. shock to EC decreases GDPC for Cape Verde. The negative response maintains a steady state after five years. In Sierra Leone, GDPC response was positive for two years and declined sharply until five years became persistent.

The negative response for The Gambia's GDPC falls until the fifth year, then increases but remain in the negative region. However, for Liberia's GDPC, responses gradually decline until the third year when it starts increasing and crossing over to the positive region. Therefore, we conclude that for Cape Verde and The Gambia, shocks to energy consumption will have a negative impact on GDPC in both the short-run and long-run, slight positive impact in the short-run and negative impact in the long run for Sierra Leone GDPC, and negative impact in the short-run and positive impact in the long-run for Liberia GDPC.

### 4.5 Variance decomposition

**Fig. 6:**
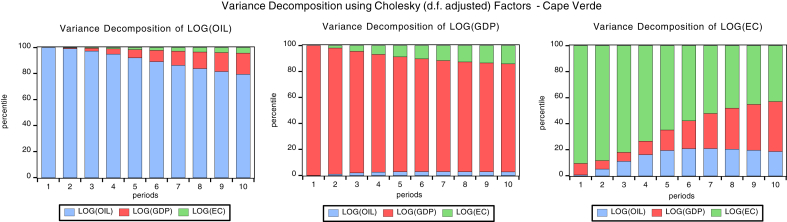
Variance decomposition for Cape Verde. Source: Authors.

**Fig. 7:**
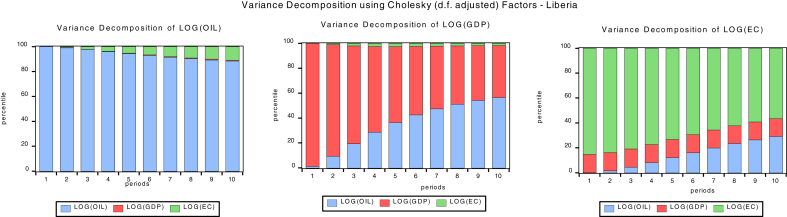
Variance decomposition for Liberia. Source: Authors.

**Fig. 8:**
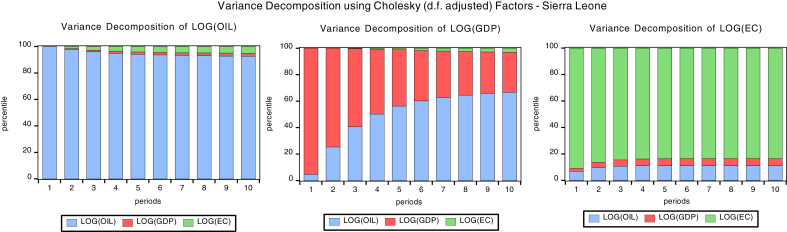
Variance decomposition for Sierra Leone. Source: Authors.

**Fig. 9:**
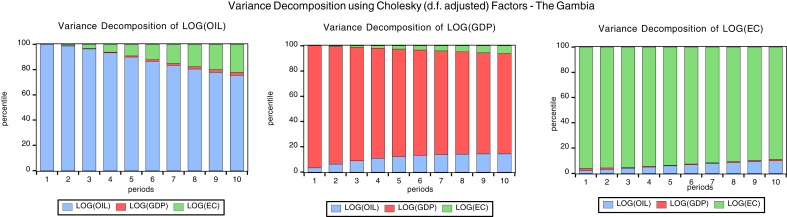
Variance decomposition for the Gambia. Source: Authors.

Variance decomposition explains the proportion of the forecast error variance that impacts its own shocks and the other variables in the unrestricted autoregressive (VAR) model. Figs. 6, 7, 8 and 9present the graph representations of variance decomposition of the global oil price shock and macroeconomic variables for the oil importing economies. We also examine the Cape Verdean economy, observing that in the short-run (year 3), shocks to energy consumption (EC), GDPC, and oil price account for 81.99%, 6.87%, 11.13% fluctuation in EC (own shock), GDPC, and global oil price respectively. In the long-run (year 10), shocks to EC, GDPC, and global contribute 42.88%, 38.41%, and 18.71% variation of the fluctuation in EC respectively. For Liberia, in the short-run, shocks to energy consumption (EC), GDPC, and global oil price account for 92.35%, 3.6%, 5.50% variation of the fluctuation in EC respectively. Hence, in the long-run, shocks to EC, GDPC, and global oil price contribute 85.83%, 3.31%, and 10.85% variation of the fluctuation in EC respectively

Sierra Leonean economy in the short-run shocks to energy consumption (EC), GDPC, global oil price account for 84.43%, 4.81%, and 10.75% variation of the fluctuation in EC respectively. Hence, in the long-run, shocks to EC, GDPC, and oil price cause 83.45%, 5.42%, and 11.12% variation of the fluctuation in EC. The Gambia in the short-run shocks to energy consumption (EC), GDPC, and oil price cause 89.92%, 5.90%, and 4.18% variation of the fluctuation in EC respectively. Hence, in the long-run, shocks to EC, GDPC; global oil price contribute 85.45%, 5.42%, and 31.99% variation of the fluctuation in EC respectively. In general, the shock to global oil price has the greatest impact on economic consumption in the short-run for Cape Verde and in the long-run for The Gambia. Also, global oil price have the least impact to EC in the short-run and long-run for Liberia.

In the short run, the shock to global oil prices, GDPC and EC causes 1.9%, 93.56% and 4.44% variation of the fluctuation in GDPC for Cape Verde, while in the long-run the shock to global oil price, GDPC and EC causes 2.72%, 83.31% and 13.96% variation of the fluctuation in GDPC. For Liberia in the short-run, the shock to global oil prices, GDPC and EC causes 17.03%, 82.77% and 0.18% variation of the fluctuation in GDPC for Cape Verde, while in the long-run the shock to global oil price, GDPC and EC causes 43.06%, 55.81% and 1.13% variation of the fluctuation in GDPC. Sierra Leonean economy in the short-run, the shock to global oil prices, GDPC and EC causes 40.65%, 59.18% and 0.16% variation of the fluctuation in GDPC for Cape Verde, while in the long-run the shock to global oil price, GDPC and EC causes 66.80%, 30.21% and 2.99% variation of the fluctuation in GDPC.

With respect to The Gambia, in the short-run the shock to global oil prices, GDPC and EC causes 5.84%, 78.51% and 15.64% variation of the fluctuation in GDPC for Cape Verde, while in the long-run the shock to global oil price, GDPC and EC causes 18.94%, 44.44% and 36.61% variation of the fluctuation in GDPC. In general, a shock to global oil price has the greatest impact to GDPC in the short-run and long-run for Sierra Leone. Also, the shock to global oil price have least impact on GDPC in the short-run and long-run for Cape Verde. In addition, the shock to EC has the greatest impact to GDPC in the short-run and long-run for The Gambia. Shocks to EC have least impact on GDPC in the short-run and long-run for Liberia.

### 4.6 Discussion of oil price implications and policy responses

The general study in energy economics debate that increasing oil prices will have an adverse effect on net oil-importers while increasing oil prices will benefit net oil-exporters (Aasim et al., 2015). The empirical results in our study under the 5 per cent significance level suggest that oil price increase will benefit GDPC in Liberia and Sierra Leone for the VAR model.

The result is in line with the findings of Abimelech et al. (2017), where oil price shocks stimulate the Liberian economy. Results for Cape Verde and Sierra Leone are insignificant, but the graphical representation of the impulse response function shows that a positive shock to global oil price will temporarily increase GDPC of Cape Verde, Liberia, Sierra Leone and the Gambia. Oil shocks to GDPC for Cape Verde, Liberia, Sierra Leone lies in the positive region in both short-run and long-run, while The Gambia lies in the negative region for all periods.

Marco (2011) shows that rising prices for other commodities has helped some oil-importing countries manage increasing oil prices. This can be the case of Cape Verde, Sierra Leone, and the Gambia, that have a positive response towards global oil price increase. Cape Verde majorly exports fuel, shoes, garments, fish, and hides; Sierra Leone majorly exports diamonds, rutile, cocoa, coffee, and fish; Liberia exports iron, diamonds, timber, rubber, cocoa, coffee. Revenues and savings from exports and falling oil prices offset the adverse impact that could occur from rising oil prices. However, in the case of The Gambia that mainly exports peanuts, fish and cotton; the low level of export of other commodities is likely not able to offset the adverse effect of the oil price increase. The Gambia need to create policies that can attract foreign direct investments and create competitive industrial production for export goods. This way the level of export for other commodities will increase, and revenues and saving will aid the government to mitigate oil price increase.

Energy shocks on net oil-importing countries have experienced challenges especially in implementing policy responses that can effectively transcend to economic growth. The stylized facts of net-oil importing countries can differ based on these factors: net import of oil per GDP, oil dependence in the energy mix, efficiency of energy production, and level of exports, revenues and savings from international trade. In order to evaluate the real effect of energy shocks in low-income West African economies, it is essential to understand that the macroeconomic framework varies for these countries and also responses to control the impact of high oil prices could differ. Government or international organisations can implement policies that can maximize the positive impacts and mitigate the negative impact based on the macroeconomic structure of the net oil importing country.

Policies can be price-based such as passing price increase and subsidies to consumers. The price increase can be passed on to consumers directly and indirectly. The government of developing net oil-importing economies that pass through prices to consumers maybe be concerned with inflationary pressures if the oil price increase is tied to the rate of inflation in these countries; therefore, tight monetary policy has to be implemented to manage inflationary pressures. This can be challenging especially for governments that want to remove subsidies to link domestic price to the global market price. This can trigger reactions from the citizens, therefore this process can be made easier by making domestic prices cheaper than international price when there is a global oil decline, then adopting a policy of gradual adjustment to complete removal of subsidy.

The governments of net-oil importing poor countries such as The Gambia, Liberia, and Sierra Leone can be challenged with the decision to increase subsidy, in order to protect household and businesses from rising oil prices. Targeted subsidy for refined oil products can be problematic to implement given that oil product such as kerosene can be easily converted to other fuels and liquid fuels are easy to smuggle across borders, which can have an adverse effect on government expenditure and the worsening of the balance of payment that consequently results to further debt crises.

Hedging product purchases using futures contracts on net oil-importing countries with low export of other commodities especially The Gambia can mitigate the adverse effect of any positive oil price shock. Oil companies that are subsidiaries of multinationals can purchase petroleum products using futures contracts when is presumed that price can increase in the future and also this aids the government to maintain a steady price for the consumers. Net oil-importing countries can diversify into alternative sources of energy in order to reduce the high dependence on oil. Some of the sources are natural gas, solar, hydro, wind, biofuel, and geothermal. Investing in cleaner energy such as biofuels can replace oil, by becoming a domestic source of energy.

## 5 Conclusion

The study explains the effects of the oil price shock on developing net oil-importing African countries, through the investigation of oil price shocks and macroeconomic relationship using an unrestricted vector autoregressive model. The study employs the Granger causality test (G-test) to examine whether past values of oil price changes can predict current changes in macroeconomic variables of the selected net oil-importing countries. The result for the G-test shows that oil prices granger causes GDPC in Liberia and Sierra Leone where the magnitude indicates a positive relationship, as oil increases the GDPC also increases. The result is consistent with the work of Abimelech et al. (2017). Oil price is observed not to Granger cause GDP per capita (GDPC) for Cape Verde and The Gambia. Moreover, energy consumption granger causes GDPC for CapeVerde and The Gambia where the magnitude of indicating a negative relationship, as energy consumption (EC) increases GDPC decreases.

The result is similar to the findings of Bekhet and Yusop (2009) on the Malaysian economy. In addition, oil price granger causes energy consumption in the Gambia where the magnitude indicates a positive relationship. The study also presents the result for a 10 years forecast period. The result shows that when a positive shock is applied to global oil prices GDPC for Cape Verde, Liberia, and the Gambia temporary decreases but with a generally positive impact in both the short-run and long-run.

The general study in energy economics debate that increasing oil prices will have the adverse effect on net oil-importers while increasing oil prices will benefit net oil-exporters (Aasim et al., 2015). We suggest that revenues and savings from high exports and falling oil prices offset the adverse impact that could occur from rising oil prices. However, for The Gambia, the impact of global oil shocks lies in the negative region both in the short-run and long-run. A major question arises from this discussion to what extent net-oil importing countries can cope with increasing oil price. This requires further studies to observe oil price shocks on other macroeconomic variables of net-oil importing countries that affect international trade such as current account balance, exchange rate and inflation.

We also observe from the impulse response function that a positive oil price shock will increase energy consumption in Cape Verde, Liberia and The Gambia in both the short-run and long-run. There is also a positive response on Sierra Leone's Energy consumption, but responses are negative in the short-run while in the long-run are persistent. However, as shocks to energy consumption increases GDP per capita of Cape Verde, Sierra Leone, and The Gambia decreases. Impulse response function indicates that for the GDPC for Cape Verde and The Gambia shocks have a negative impact both in the short-run and long run. Sierra Leonean GDPC indicates slight positive impact in the short-run and negative impact in the long-run, while for Liberia negative impact in the short-run and positive impact in the long-run for Liberia.

The study highlights that the stylized facts of net-oil importing countries can differ based on the following factors: net import of oil per GDP, oil dependence in the energy mix, efficiency of energy production, and level of exports, revenues and savings from international trade. Therefore, it is essential to understand that the macroeconomic framework of an economy, before implementing policies that can effectively mitigate the adverse effect of the oil price increase through government initiative or foreign aid. We recommend policies governments can implement such as passing price increase and subsidies to consumers, hedging using futures contracts, reducing high dependence on petroleum and petroleum products by investing in an alternative sources of energy.

The government can pass price increase to consumers by adjusting prices when international spot prices change using a certain percentage tied to the local currency. Domestic prices are naturally adjusted due to foreign exchange volatility, global oil price fluctuations or both. This strategy protects consumers from minor changes but can be inefficient in the case of large fluctuations. Therefore, major oil price shocks can be passed on to the end-consumer.

Government that wants to completely pass-through prices to consumers are concerned with inflationary pressures. This is a major issue because inflation will need to be controlled therefore tight actions over monetary policy is essential. This problem can be severe for governments that wants to cut subsidies and adjust prices at the international market level. The value of the subsidy for different oil products has to be decided based on the macroeconomic framework of the country. For example, country like Sierra Leone that wants to encourage the agricultural sector will decide to subsidize diesel products totally or partially to limit the effect of increasing prices on diesel imports. In addition, the government will also subsidize kerosene for the low-income households, in order to improve the standard of living.

Policies to reduce the cost of import such as hedging can help mitigate the adverse effect of oil price increase. Commercial oil companies that are subsidiaries of multinationals can purchase petroleum products using futures contracts when is presumed that price can increase in the future this can help the government to maintain a steady price for the consumers. The government can diversify into non-petroleum sources of energy to reduce the high dependence on oil. The prominent non-petroleum sources are natural gas, biomass and renewable energy sources such as solar, geothermal, wind, and hydro.

## Declarations

### Author contribution statement

Obindah Gershon: Conceived and designed the analysis; Wrote the paper.

Nnaemeka Emmanuel Ezenwa: Analyzed and interpreted the data; Wrote the paper.

Romanus Osabohien: Contributed analysis tools or data; Wrote the paper.

### Funding statement

This work was supported by Covenant University Centre for Research Innovation and Discover (CUCRID).

### Competing interest statement

The authors declare no conflict of interest.

### Additional information

No additional information is available for this paper.
